# Auditory Brainstem Implant: surgical technique and early audiological results in patients with neurofibromatosis type 2

**DOI:** 10.1016/S1808-8694(15)31371-9

**Published:** 2015-10-17

**Authors:** Ricardo Ferreira Bento, Rubens Vuono Brito Neto, Robinson Koji Tsuji, Marcos Queiroz Telas Gomes, Maria Valéria Schmidt Goffi-Gomez

**Affiliations:** 1 Full Professor of Otorhinolaryngology at Faculdade de Medicina da USP; 2 Associate Professor of Otorhinolaryngology at Faculdade de Medicina da USP; 3 Graduate student, assistant-physician at the Otorhinolaryngology Clinic at HC-FMUSP; 4 Assistant-physician in the course of Neurosurgery at Faculdade de Medicina da USP; 5 PhD. Speech and hearing therapist at the Otorhinolaryngology Clinic at HC-FMUSP. Course of Otorhinolaryngology at Faculdade de Medicina da Universidade de São Paulo

**Keywords:** surgery, auditory brain stem implantation, neurofibromatosis type 2, results

## Abstract

Auditory Brainstem Implants were developed to partially restore the hearing capabilities of patients without cochlear nerves bilaterally.

**Aim:**

this paper aims to discuss the clinical and surgical findings of four ABI patients.

**Materials and method:**

four patients diagnosed with bilateral schwannomas received auditory brainstem implants (ABI) and had one of their tumors resected in the same surgical procedure. Clinical aspects, surgical technique, anatomic landmarks, and outcomes were analyzed.

**Results:**

the anatomic landmarks were identified in all four patients in relation to the foramina of Luschka. Two patients had CSF leaks. The electrodes were well positioned and hearing sensation was good enough to allow for sound recognition and assist patients perform lip reading.

**Conclusion:**

the outcomes observed in our patients were quite encouraging and offer great perspectives for those suffering from deep bilateral deafness and impaired central auditory pathways.

## INTRODUCTION

Auditory Brainstem Implants were developed to partially restore the hearing capabilities of patients without cochlear nerves bilaterally^1^. They were first developed as a one-channel electrode at the House Ear Institute (HEI), in Los Angeles, California. This first model was implanted in 25 patients between 1979 and 1992, producing precarious clinical results^2^. A multichannel device was then developed from this experience by HEI in partnership with the Cochlear Corporation (Englewood, Colorado) and the Huntington Medical Research Institute (Pasadena, California). The device was made up by an internal unit containing a silicone cable with disc-shaped platinum electrodes and an interface to a Nucleus Mini-22 speech processor.

Patients classically benefitting from this electronic, surgically-implanted hearing aid are those diagnosed with neurofibromatosis type 2 (NF-2), bilateral vestibular schwannomas, and children with cochlear nerve congenital aplasia. Today the indication for auditory brainstem implants has been extended to patients with damaged VIII nerves and those who are ineligible for conventional cochlear implants such as post-meningitis patients with ossified cochleas^3^, ^4^. Although the first implantation procedure dates back from 1979, only in October of 2000 was it approved for clinical use by the Food and Drug Administration.

## OBJECTIVES

This paper aims to discuss the clinical and surgical findings pertaining to the first four ABI patients treated in our institution, looking at indication criteria, surgical technique, and outcomes.

## MATERIALS AND METHOD

Four patients diagnosed with bilateral vestibular schwannomas received auditory brainstem implants (ABI) and had one of their tumors resected in the same surgical procedure. This study was approved by the medical ethics committee at our institution under permit M.16-196/2007.

The translabyrinthine approach was chosen to remove the tumors and expose the cochlear nerve on the lateral wall of the IV ventricle (foramina of Luschka). Each patient was implanted one Cochlear N24® ABI internal unit. The VII, IX, X and XI nerves were monitored through continuous electrophysiology during surgery. Electric response brainstem audiometry was used to check whether the electrodes had been properly put in place.

Free field audiometry tests were conducted to check patient hearing after three months of activation.

**Figure 1:**
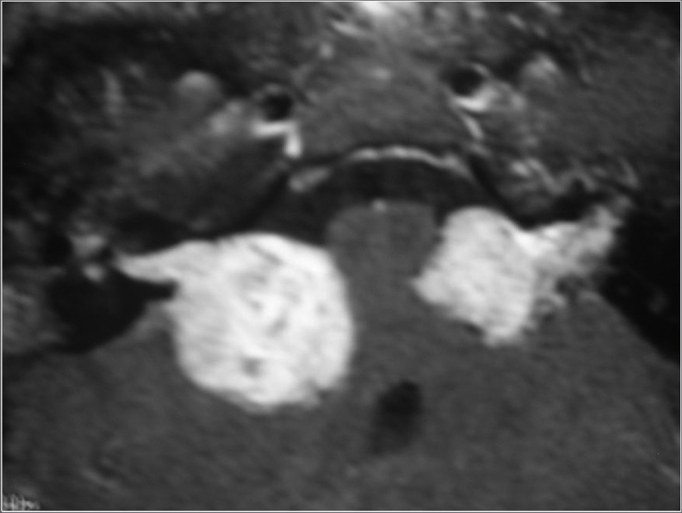
MRI (T1 contrast-enhanced) case 1 preoperative axial view.

**Figure 2:**
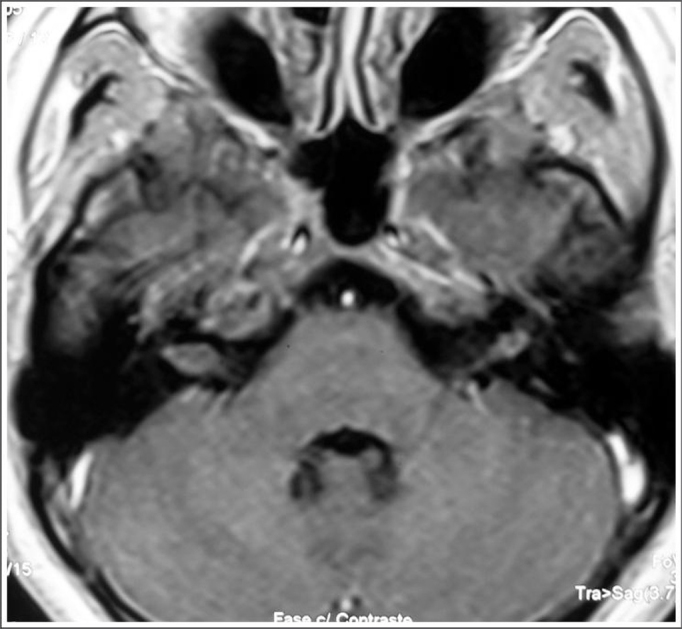
MRI (T1 contrast-enhanced) case 2 preoperative axial view.

**Figure 3:**
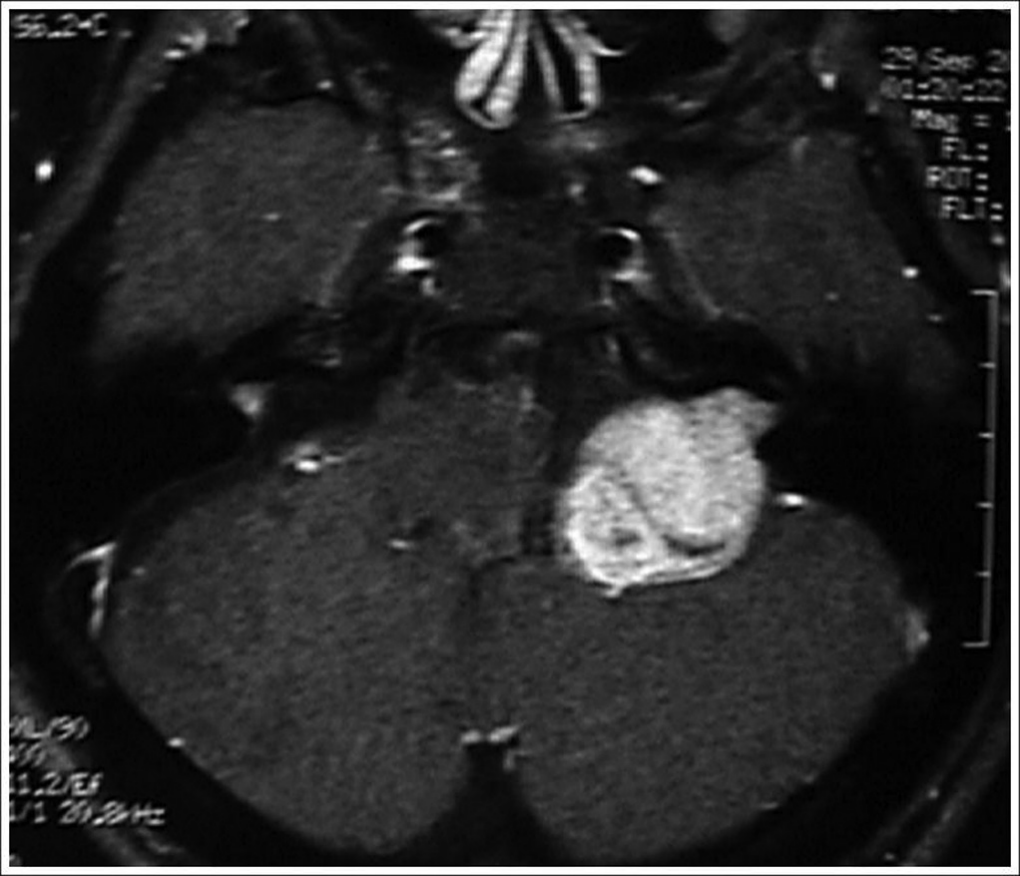
MRI (T1 contrast-enhanced) case 3 preoperative axial view.

**Figure 4:**
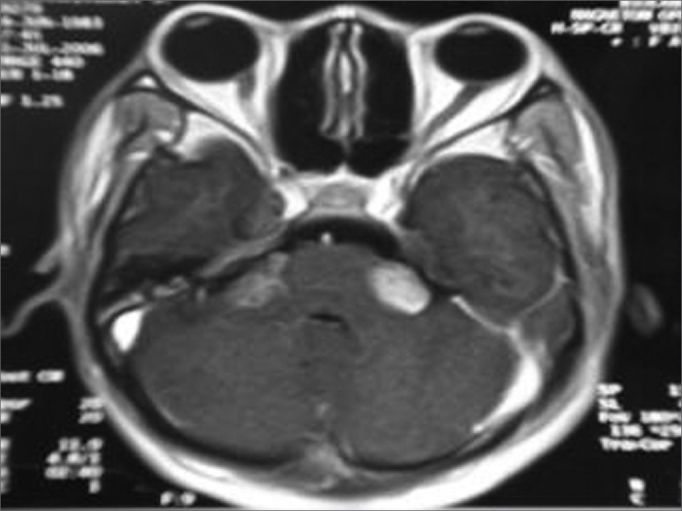
MRI (T1 contrast-enhanced) case 4 preoperative axial view.

Patient, surgery, and external unit activation data can be seen on Table 1. Figure 1, Figure 2, Figure 3, Figure 4 show X-ray images of the removed tumors.

**Table 1:** Patients, surgery, and external unit activation data.

Patient	Age	Gender	Diagnosis	Tumor Size	Surgery Date	Activation Date
1	28	M	NF2	3,5 cm	Jan 2006	Feb 2006
2	25	F	NF2	IC	Mar 2006	Apr 2006
3	25	M	NF2	4.0 cm	Mar 2006	Apr 2006
4	26	M	NF2	2.0 cm	Dec 2006	Jan 2007

## RESULTS

The classic translabyrinthine approach was satisfactorily used to remove the tumors and identify the foramina of Luschka. The anatomic landmarks in relation to the foramina, namely the IX nerve and the cerebellar floculus, were successfully accessed by the surgeon by completely uncovering the jugular bulb from the bony layer that wraps it and completely exposing the entire dura from the middle and posterior cerebral fossae. The dura in the posterior fossa was broadly opened. The incision had borders close to the jugular bulb, the sigmoid sinus, and the upper petrous sinus. All procedures were uneventfully completed, and the electrodes were positioned touching the cochlear nucleus on the lateral wall of the IV ventricle. Cerebrospinal fluid leaks occurred in patients 2 and 3. They were treated with lumbar punction (patient 3) and obliteration surgery using abdominal fat (patient 2).

**Graph 1:**
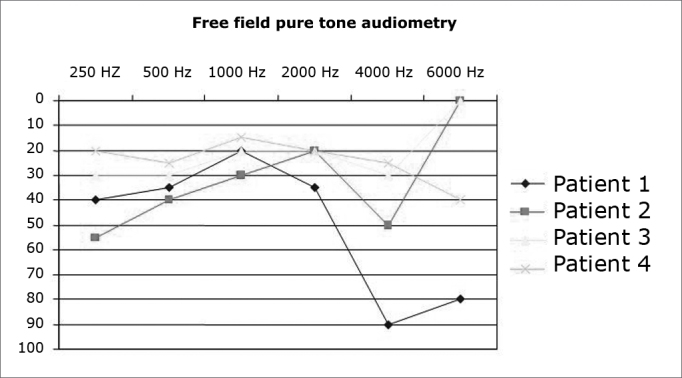
Hearing thresholds of ABI patients three months after activation

Activation was carried out on average at 30 days after surgery, with the patients in a surgical setting with cardiac monitoring. Except for patient 1, who experienced sound sensation in only one electrode, all others made reference to hearing sensation in more than 12 electrodes. Side effects were observed in 88% of the upper, lower limbs, and abdomens and in 12% of all heads and necks (throat and nausea). Graph 1 shows the hearing outcomes as observed in tone free field audiometry tests carried out three months after activation.

## DISCUSSION

The concepts related to auditory brainstem implants are similar to those applicable to the currently available cochlear implants, except for the electrode setup which, in the first case, is designed to be introduced at the level of the cochlear nerve and not at the cochlear tympanic ramp. Patients who for functional or anatomic reasons are unable to receive electric stimuli in their inner ears are the best candidates for this treatment. In more socially advanced countries, the main cause of bilateral structural loss of peripheral auditory pathways is neurofibromatosis type 2, a condition that typically evolves into bilateral vestibular schwannomas^5^. That however is not the case for Brazil. Infection is still the main etiology for deafness, meningitis certainly being the main culprit^6^. Some 23.9% of all cochlear implant patients experienced hearing loss following meningitis. This is a reason for great concern, once the hearing prognosis after implantation is closely connected to the number of viable neural elements and the accurate positioning of the electrodes in the cochlea. And meningitis goes against both factors. Firstly, it is the etiology that most destroys cochlear hair cells and cochlear nerve neurons; secondly, it will often lead to some degree of ossification in the otic capsule. Apart from the functional involvement, meningitis was also responsible for all six cases of failed electrode positioning during surgery, with consequent removal of the internal unit and a second procedure to implant new electrodes. This infection only comes to greatly increase the need for an alternative to conventional cochlear implants in Brazil. Neurofibromatosis type 2 patients are rare, even at reference care centers. Nonetheless, when taking into account the tragic, progressive slow evolution of this disease, the impact for the patients of having some of his or her hearing restored is extremely relevant.

In our few first cases, we tried to perform surgery on patients with the so-called classic indication for both tumor removal and electrode implantation. Patient 1 was first selected for having extensive knowledge on the procedure, as he has for years informally studied NF2 and gone through a number of surgical procedures previously. Full awareness of the possibilities and limitations of the proposed procedure is a key factor in fine-tuning the patients’ expectations and consequent post-operative satisfaction. Both patients and their families must be well-informed of the natural history of NF2, the surgical procedure itself, and of the possibility of there being partial hearing restoration after implantation. Patients and families go through a rigorous psychological and social evaluation, not to mention medical and audiological screening.

There are two main approaches for auditory brainstem implant placement: suboccipital retrosigmoid and translabyrinthine^7^. The elected approach should provide for ample enough visualization to allow for the precise identification of the anatomic landmarks used as reference for accurate electrode placement. Surgeons base their choice on their own personal experience. Most ENT surgeons pick the translabyrinthine approach to remove large vestibular schwannomas or when treating patients with deteriorated hearing. As tumors had to be removed and electrodes implanted on one same surgical procedure, this was the approach we chose. The surgical technique used to place the electrodes is similar to the one used to remove vestibular schwannomas. The cochlear nucleus complex, made up by the ventral and dorsal cochlear nuclei, is the site for electrode placement. The ventral cochlear nucleus is the main nucleus for neural impulse transmission from the VIII nerve, and its axons form the main ascending pathway of the cochlear nerve. Neither the ventral nor the dorsal nucleus can be seen during surgery, and their location can only be inferred from the identification of anatomic landmarks adjacent to them. The lateral end of the IV ventricle, the foramina of Luschka, is located between the outputs of the glossopharyngeal and facial nerves. As the floculus is moved away, the surgeon can visualize a depression between the above mentioned cranial nerves, which is the site where the electrode is to be inserted. Usually only one stump of the cochlear nerve can be identified. It may also be used as a landmark for the lateral recess. One should bear in mind that the removal of large tumors often introduces changes to the anatomy of the area, mainly in relation to the emergence of the VIII nerve from the pons which becomes hard to identify as the integrity of the nerve is lost during surgery and remains of the arachnoid are still present. Observation of the anatomically preserved structures, besides the distal and proximal tumor site, and intraoperative electromyography are undoubtedly useful parameters during electrode placement. In our four cases the anatomic landmarks were well preserved, as seen mainly in the first two patients whose tumors were smaller. Patient 1 had a small tumor that did not project itself into the posterior cerebral fossa, thus leaving the surface of the brainstem intact and, therefore, with easily identifiable landmarks. The main landmarks for patient 2 were the bulb cranial nerves and the cerebellar floculus, with a well-preserved foramina of Luschka.

Patient 2 had bilateral intracanal lesion and recent deep dysacusis, turning her into an excellent candidate for the first case, both from the surgical standpoint, as the anatomic landmarks for the foramina of Luschka would not be deformed by the tumor, and for her improved hearing prognosis due to the recent onset of sensorial deprivation. Although this analysis stood correct, the patient evolved to a rather uncommon condition after surgery, as she had a CSF leak that did not respond to a lumbar shunt. She thus had to be operated again to surgically close the fistula. Her routine CSF test showed tumor cells further described by the pathologist as coming from a choroid plexus papilloma. The aggressive evolution of the tumor and the consequent need for chemotherapy and radiotherapy led the patient to stop using the implant during treatment. Today the patient is stable and clinically well, and went back to using the implant daily. Metastasis in the inner ear meatus is rarely observed, and seldom can it be radiologically differentiated from small vestibular schwannomas. This rare differential diagnosis is something we should all have in mind, as one of the approaches for small ear tumors is clinical follow-up.

Patients 3 and 4 had larger tumors, thus making it harder to identify the foramina of Luschka as anatomic changes were present in the area. Once it was found, the electrodes could be well positioned in both cases.

The hearing outcomes for all four patients were quite encouraging. In our sample, the presence of hearing thresholds corresponding to mild hearing loss and even normal thresholds in some frequencies suggest the patients were able to perceive many sounds in the environment, including high frequencies, showing that the ability to perceive environment sounds and perform speech recognition assisted by lip reading can be restored with this type of implant^8^, ^9^. Although the outcomes provided by the auditory brainstem implants were compared against those of one-channel cochlear implants^10^, the early free field audiometry test results were positively surprising, mainly in terms of patient compliance and daily use of the hearing aid.

## CONCLUSION

In spite of the early-stage results gathered, we believe the outcomes observed in our patients were quite encouraging and offer great perspectives for those suffering from deep bilateral deafness and impaired central auditory pathways.
